# Prescription and non-prescription antibiotic dispensing practices in part I and part II pharmacies in Moshi Municipality, Kilimanjaro Region in Tanzania: A simulated clients approach

**DOI:** 10.1371/journal.pone.0207465

**Published:** 2018-11-21

**Authors:** Pius G. Horumpende, Tolbert B. Sonda, Marco van Zwetselaar, Magreth L. Antony, Filemon F. Tenu, Charles E. Mwanziva, Elichilia R. Shao, Stephen E. Mshana, Blandina T. Mmbaga, Jaffu O. Chilongola

**Affiliations:** 1 Department of Biochemistry and Molecular Biology, Kilimanjaro Christian Medical University College, Moshi, Tanzania; 2 Kilimanjaro Clinical Research Institute, Moshi, Tanzania; 3 Department of Preventive Medicine and Research, Lugalo General Military Hospital and Military College of Medical Sciences (MCMS), Dar es Salaam, Tanzania; 4 Section of HIV Viral Load and Early Infant Diagnostics, National Health Laboratory Quality Assurance and Training Centre, Dar es Salaam, Tanzania; 5 Department of Microbiology and Immunology, Catholic University of Health and Allied Sciences, Mwanza, Tanzania; Natural Environment Research Council, UNITED KINGDOM

## Abstract

Antibiotic dispensing without a prescription poses a threat to public health as it leads to excessive antibiotic consumption. Inappropriate antibiotic availability to the community has been documented to be amongst drivers of antimicrobial resistance emergence. Community pharmacies are a source of antibiotics in low and middle-income countries (LMICs). We aimed at assessing antibiotic dispensing practices by community pharmacy retailers in Moshi urban, Kilimanjaro, Tanzania and recommend interventions to improve practice. Using a Simulated Client (SC) Method, an observational cross-sectional survey of antibiotic dispensing practices was conducted from 10^th^ June to 10^th^ July 2017. Data analysis was done using Stata 13 (StataCorp, College Station, TX, USA). A total of 82 pharmacies were visited. Part I pharmacies were 26 (31.71%) and 56 (68.29%) were part II. Overall 92.3% (95% CI 77.8–97.6) of retailers dispensed antibiotics without prescriptions. The antibiotics most commonly dispensed without a prescription were ampiclox for cough (3 encounters) and azithromycin for painful urination (3 encounters). An oral third generation cephalosporin (cefixime) was dispensed once for painful urination without prescription by a part I pharmacy retailer. Out of 21, 15(71.43%) prescriptions with incomplete doses were accepted and had antibiotics dispensed. Out of 68, 4(5.9%) retailers gave instructions for medicine use voluntarily. None of the retailers voluntarily explained drug side-effects. In Moshi pharmacies, a high proportion of antibiotics are sold and dispensed without prescriptions. Instructions for medicine use are rarely given and none of the retailers explain side effects. These findings support the need for a legislative enforcement of prescription-only antibiotic dispensing rules and regulations. Initiation of clinician and community antibiotic stewardship and educational programs on proper antibiotic use to both pharmacists and public by the regulatory bodies are highly needed.

## Introduction

The ability to acquire antibiotics from a community pharmacy without a prescription poses a challenge in the control of irrational antibiotic consumption. There is a relationship between irrational antibiotic consumption and development of antibiotic resistance.[1–3] Excessive antibiotic consumption leads to antimicrobial resistance through a postulated mechanism of antibiotic selection pressure.[4] Other factors responsible for development of antibiotic resistance include acquisition of resistance genes from the environment (plasmids) and bacterial mutations.[5] As mutation is a natural phenomenon we cannot control or influence its occurrence. However, we can mitigate the rapid development of antibiotic resistance by employing a regulated and a prudent use of antibiotics.[6] Antibiotic resistance is escalating and has recently been declared by WHO as a world crisis.[7] Amongst the consequences of antibiotic resistance include severe infections, longer hospital stays, disease complications and increased morbidity and mortality.[8]

A recent systematic review and meta-analysis on global access to antibiotics without prescription in community pharmacies indicates that non-prescription supply of antibiotics is highest in South America.[9] There is an overall global increase in antibiotic consumption but notably more than three-quarters of the overall increase in antibiotic consumption occurred in Brazil, Russia, India, China, and South Africa.[10] In LMICs rules, regulations and legislation may be in place but there is a lack of enforcement.[11] The readily available antibiotics over the counter leads to excessive and hence inappropriate consumption.[12]

In most countries, antibiotics are accessed through community pharmacies.[13] In Africa and other LMICs, community pharmacies often act as the first point of contact with health care.[14,15] Pharmacy staff in these countries need to have the basic training requirements and be regularly trained in dispensing skills.[16–18] In these countries community antibiotic consumption is high, posing a risk of development of community antibiotic resistance.[2] The poor health care delivery systems in LMICs influence people to resort to community pharmacies for treatment[19] without seeking medical consultation.

In Tanzania although antibiotics are classified as prescription only drugs, the sale and dispensing of antibiotics without prescription is common.[20] Several reports have been published as initiatives towards advocacy on rational antibiotic consumption and the consequences of irrational antibiotic consumption. A situational analysis done in Tanzania (GARP) in 2015 proposes rationalizing antibiotic use and reducing the widespread inappropriate antibiotic dispensing.[21] In 2016 an analysis on strengths, weaknesses, opportunities and challenges (SWOC) for developing antimicrobial stewardship programs in Tanzania reiterated increasing investments in medicines regulatory authority and strengthening advocacy on rational use of antimicrobials.[22] In August 2017 the Tanzania’s Ministry of Health Community Development Gender Elderly and Children (MoHCDGEC) launched The Tanzania National Action Plan (2015–2022) to combat antimicrobial resistance.[23]

The current study aimed at determining the extent of non-prescription antibiotic dispensing practices, dispensing of incomplete antibiotic doses and dispensing of antibiotics to two days’ duration of ailments such as fever, diarrhoea, cough painful urination and runny nose in Moshi Municipality in Kilimanjaro Region, Tanzania to recommend plausible interventions to improve antibiotic dispensing practices.

## Materials and methods

### Study settings, design, and population

A simulated client, cross-sectional study was done between 10^th^ June and 10^th^ July 2017. It involved drug retailers (dispensers) in community (part I and part II) pharmacies in Moshi Municipality in Kilimanjaro, North-Eastern Tanzania. Moshi urban with a population of 184,292 inhabitants, is the administrative center of the Kilimanjaro Region with a total population of 1,640,087 million inhabitants.[24] These community pharmacies were categorized as part I and II according to Tanzania Food and drug Authority (TFDA) classification system. TFDA is responsible for licensing, monitoring and regulating food, drugs and cosmetics in Tanzania. A part I pharmacy is a fully-fledged pharmacy unit operating under the direct supervision of a registered pharmacist. In these premises, a pharmaceutical technician, a pharmaceutical assistant and other drug dispensers assist a pharmacist. A part II pharmacy is a facility that sells drugs that appear in the schedule of part two poisons list of the -TFDA Act of 2003.[25,26] A supervisor of part II pharmacy is any pharmaceutical personnel (that is: a Pharmaceutical technician or a pharmaceutical assistant) or any person who has had a five weeks’ training on Accredited Drug Dispensing Outlets (ADDO) training with a certificate hanging inside the shop in a visible area. ADDO training equips a supervisor with rational dispensing knowledge and skills. All pharmacies in Tanzania are jointly regulated by the Pharmacy Council and the Tanzania Food and Drug Authority (TFDA). The differences between part I and II pharmacies include the fact that the category of drugs permitted in part I are more diverse in terms of strength and variety. These drugs are ‘reserved’ as last resort in severe and life threatening infections than those in part II. As an example cephalosporins and carbapenems are allowed in part I but not allowed in part II pharmacies. The second difference is in the supervision aspect. Part I pharmacies are supervised by a registered pharmacist on site while a pharmaceutical technician/assistant or an ADDO trained person can supervise part II pharmacy. The third difference entails premises’ requirements. A part I pharmacy premises require an air-condition, refrigeration of drugs and a dispensing window for antibiotics.

### Pharmacy sampling and study procedures

A list of registered retail community pharmacies obtained from the Kilimanjaro Regional Pharmacist indicated that by June 2017 there were 26 part I retail pharmacies and 116 part II retail pharmacies. All registered part I pharmacies were surveyed. Randomness was determined for part II pharmacies by simple random sampling. In total there were 116 part II pharmacies. Each pharmacy was assigned to an envelope bearing a number with its name inside. Envelopes were serially numbered. Microsoft—Excel software computer program was used to randomize the envelope numbers to obtain the names of the 56 part II pharmacies. In total, 82 pharmacies were visited by the study’s simulated clients (SC). Five SC visited pharmacies with common complaints of runny nose, unspecified fever, cough without fever or hemoptysis, acute watery diarrhea without fever, blood, pus, nausea, or vomiting and a young woman with painful urination, urinary frequency (every two to three hours), without fever, flank pain, nausea, vomiting, or urethral discharge. Each of the SC had one of the symptoms of cough, fever, diarrhoea, runny nose and pain during urination. The SC and their number of simulations were cough (16) diarrhoea (15) fever (13) runny nose (22) and pain during urination (16). The duration of all symptoms was for two days. On presenting a prescription to the retailer, a SC asked for medication as prescribed. In case the SC had no prescription he or she would state the symptom and ask for medicine from the retailer. The pharmacy retailer had a decision to offer or deny medication with or without giving a reason. In case medication was dispensed, the SC would wait for instructions to use the medicine and, if instructions were not voluntarily given, the SC would probe them from the retailer. The SC would again probe for medication side effects if the retailer did not voluntarily explain them. Retailers in the pharmacies were not aware of the study and were made to believe they were treating real patients.

The SC had prescriptions on the first day and had no prescriptions on the second day. Each SC was given enough money by the principle investigator for buying medicines if the retailer was willing to sell. The SCs had to ask for the receipt as a proof that they actually bought the medicine in case they were not denied one. A structured questionnaire to capture data within fifteen minutes after the visit to the pharmacy and out of view of the pharmacy staff was given to each SC. Training was performed to make sure SCs understood their tasks. A pilot study was conducted prior to data collection in ten similar pharmacies in Moshi, which were not included in the study.

### Data analysis

Data were double entered and managed using Open-Clinica (Open-Clinica LLC, MA, US). Data analysis was performed using Stata 13 (StataCorp, College Station, TX, USA). All analyses were descriptive. Results were summarized as frequencies (percentages).

### Ethics and confidentiality

Ethical clearance was obtained from Kilimanjaro Christian Medical University College Research Ethical Committee with certificate number 892. Permission to conduct this study was granted by Kilimanjaro Regional Administrative Secretary. Neither individual pharmacy nor retailer’s identities were recorded or disclosed. The purpose of the study was explained to the owners of retail pharmacies six months before the study period and informed consent was obtained. Pharmacy owners were informed that simulated clients would visit their pharmacies within the next six months seeking medications for various ailments and consented to participate in the study.

## Results

A total of 82 pharmacies were studied. Out of 43, 22 (51.2%) of prescriptions had complete doses and 21(48.8%) were prescriptions with incomplete doses. Out of 21,l5 (71.4%) of prescriptions with incomplete antibiotic doses were dispensed. Out of 69, 4(5.9%) retailers explained instructions for medicine use voluntarily. None of the retailers explained the drug side effects voluntarily (Table 1).

**Table 1:** Antibiotic dispensing practices in Moshi Municipality, Kilimanjaro Region, Tanzania.

Category Assessed	All responses (N)	Variable	Encounters (n)	Percentage (%)
All pharmacies	82		82	100.0
Pharmacy category	82	Part I	26	31.7
		Part II	56	68.3
Prescription status (given/not given)	82	No	39	47.6
		Yes	43	52.4
Type of dose given to those with prescription	43	Incomplete	21	48.8
		Complete	22	51.2
Medication dispensed	82	No	13	15.9
		Yes	69	84.1
Reasons for not dispensing medication^a^	13	No prescription	3	23.1
		Medicine not available	5	38.5
		Wrong indication	5	38.5
Alternative medication given against prescriptions with complete dose types	21	No	16	76.2
		Yes	5	23.8
Reasons given for dispensing alternative medication	5	No	0	0.0
	5	Yes	5	100.0
Instructions for medicine use for clients given medication^b^	69	Voluntary	4	5.8
		After probing	65	94.2
Side Effects explained^b^	69	Voluntary	0	0.0
		After probing	69	100.0
Oral antibiotics dispensed for 2 days instead of 5–7 (Incomplete doses)	21	No	6	28.6
		Yes	15	71.4

^a^ 26 encounters had medication without prescription thus reasons for not dispensing medication is not applicable.

^b^ 13 encounters were not given medication thus neither instructions for medicine use nor side effects explained are not applicable.

Amoxycillin (1 encounter) and ampiclox (3 encounters) were dispensed without prescriptions for cough in both part II and I pharmacies. Trimethoprim—Sulphamethoxazole was dispensed (2 encounters) for fever from a part II pharmacy. Cefixime (an oral third generation cephalosporin) was dispensed once for painful urination in part I pharmacy (Table 2).

**Table 2:** Antibiotics dispensing encounters without prescription by pharmacy category.

Pharmacy category^a^	Antibiotic	Symptom				
		Cough	Fever	RN ^b^	Diarrhoea	PU^c^
Part I	Amoxycillin	1				
	Ampiclox	1				
	TMX/SMX					
	Azithromycin					3
	Metronidazole					
	Erythromycin					
	Cefixime					1
	Ciprofloxacin					
	Ecofloxacin					
	Levofloxacin					
Part II	Amoxycillin					
	Ampiclox	2				
	TMX/SMX		2			
	Azithromycin					
	Metronidazole				2	
	Erythromycin					
	Cefixime					
	Ciprofloxacin					1
	Ecofloxacin					1
	Levofloxacin					1

^a^Pharmacy category I and II

^b^Runny Nose

^c^Painful Urination

Amoxyclav was dispensed (1 encounter each) for cough from parts I and II pharmacies. Azithromycin (4 encounters) and Erythromycin (1 encounter) were dispensed for fever in a part II pharmacy with a prescription. Metronidazole was dispensed for diarrhoea from both Part I (1 encounter) and II (8 encounters) pharmacies with a prescription (Table 3).

**Table 3:** Antibiotics dispensing encounters with prescription by pharmacy category.

Pharmacy category^a^	Antibiotic	Symptom				
		Cough	Fever	RN^b^	Diarrhoea	PU^c^
Part I	Amoxyclav	1				
	Azithromycin					
	Erythromycin					
	TMX/SMX			3		
	Metronidazole				1	
	Ciprofloxacin					5
Part II	Amoxyclav	1				
	Azithromycin		4			
	Erythromycin		1			
	TMX/SMX			5		
	Metronidazole				8	
	Ciprofloxacin					1

^a^Pharmacy category I and II

^b^Runny Nose

^c^Painful Urination

In part I pharmacies none of the retailers explained antibiotic side effects voluntarily. In part II pharmacies out of 53, 4(7.5%) of the retailers gave instructions for medicine use voluntarily (Table 4).

**Table 4:** Practice of antibiotic dispensing.

Variable	Pharmacy category	
	Part I (N = 26)	Part II (N = 56)
	n (%)	n (%)
Dose type		
incomplete	8 (50.0)	13 (48.1)
complete	8 (50.0)	14 (51.9)
Instructions for Medicine Use		
voluntarily	3 (15.0)	4 (7.5)
after probing	17 (85.0)	49 (92.5)
Side effects Explained		
voluntarily	0 (00.0)	1 (1.9)
after probing	20 (100.0)	52 (98.1)

Overall proportion of antibiotic dispensing encounters without prescriptions was 92.3% (77.8–97.6). Simulated clients with fever, diarrhoea and runny nose had 100% access to antibiotics without prescription (Table 5).

**Table 5:** Proportion dispensing antibiotics without prescriptions.

Symptom	Proportion (%)	95% CI
Over all	92.3	77.8–97.6
Fever	100.0	-
Diarrhoea	100.0	-
Runny nose	100.0	-
Painful urination	88.8	37.5–99.1
Cough	75.0	27.5–95.9

Of the two pharmacy types, type I pharmacy dispensed antibiotics at a frequency of 18 (26.5%) compared to type II with a frequency of 50 (73.5%) (p = 0.0004).

On disaggregating by pharmacy category the frequency of encounters of the antibiotics dispensed without prescription in part I pharmacies were 8(44.4%) while in part II pharmacies were 28(56%). The frequency of encounters of incomplete antibiotic dose dispensed from part I pharmacies were 5(50%) while those in part II pharmacies were 12(54.6%) (Table 6).

**Table 6:** Comparison of the frequency of encounters of antibiotics dispensed in Part I and Part II pharmacies in Moshi Town.

Variable	Part I Pharmacy (N = 26)			Part II Pharmacy (N = 56)			
	n	%	95% CI	n	%	95% CI	p
Medicine dispensed	18	26.5	0.06–0.48	50	73.5	0.62–0.86	0.0004
Medicine not dispensed	8	57.1	0.23–0.91	6	42.9	0.03–0.83	0.6041
Medicine dispensed without prescription	8	44.4	0.09–0.78	28	56.0	0.38–0.74	0.5485
Medicine dispensed with prescription	10	55.6	0.25–0.87	22	44.0	0.23–0.65	0.528
Incomplete dose medication dispensed	5	50	0.06–0.94	12	54.6	0.27–0.83	0.8506
Complete dose medication dispensed	5	50	0.06–0.94	10	45.5	0.15–0.77	0.8837

## Discussion

Using a SC method, we set out to describe the antibiotic non—prescription sale and dispensing behavior and the quality of antibiotic dispensing in Moshi urban pharmacies. Antibiotics can easily be obtained without a prescription in up to 92% of pharmacies surveyed in Moshi municipality. Our data show inappropriate antibiotic dispensing which predispose the public to not only increased cost of health care, but also untoward drug side effects and community antimicrobial resistance.[27] The findings of this study add to a severe paucity of data on antimicrobial consumption in LMICs, Tanzania inclusive[11] and provide an insight on empirical knowledge and actual practices of retailers in dispensing antibiotics in community pharmacy settings. Our data show a poor quality of community pharmacy practice due to non-prescription dispensing of antibiotics, lack of instructions on how to take drugs and not explaining side effects to clients. The overall antibiotics dispensed without prescription was 56%. Part I pharmacies dispensed antibiotics without prescription by 8(44.4%) while it was 28(56%) for part II pharmacies. It is clear that much effort to address the adherence to antibiotic dispensing rules and regulations should be directed to part II pharmacies. A study in Dar es Salaam found the non -prescription dispensing of medicines in private pharmacies to be 71% and 60% were prescription only medicines including antibiotics.[28] Our data therefore indicate that antibiotic non—prescription dispensing in Tanzania remains a problem requiring attention.

The proportion of dispensing an antibiotic without any prescription for cough was high (75%). Cough is a symptom of many organ systems such as respiratory, cardiovascular and renal systems. Ideally the cause of cough should be identified. In the event cough and fever are present together we clinically suspect an infection though determination of an infective aetiology as most causes of cough are viral in aetiology.[29] Having a cough and directly accessing antibiotics in a tuberculosis endemic region carries a risk of delay in proper tuberculosis diagnosis and treatment with consequent complications and death.[30,31] In most cases cough and runny nose are due to viral infection and there was no rationale for antibiotic use.[32] It is therefore inappropriate and poor pharmacy practice to sell or dispense an antibiotic for cough, a symptom with many causes. One of the consequences of excessive antibiotic consumption is bacterial antibiotic resistance. We observe that penicillin resistance gradually increased from 6% in a study from South Africa[33] to 43% in a Ghanaian study.[34] In Tanzania we observe a rising trend of antibiotic resistance especially of the phenotype Extended Spectrum Beta Lactamase (ESBL) producing gram-negative bacteria in the city of Dar es Salaam.[35–38] A similar trend is observed in studies from another north western city of Mwanza in Tanzania.[39–41] Such trends of antibiotic resistance are envisaged to spread to other cities of Tanzania should the current antibiotic non-prescription community pharmacies dispensing behavior remain unchecked.

### Antibiotic dispensing practices

Although runny nose is a predominantly viral infection,[32] our data show that an antibiotic trimethoprim-sulphamethoxazole was dispensed with prescription for runny nose by a proportion of 100% from both parts I and II pharmacies. Dispensing antibiotics for runny nose connotes inappropriate antibiotic use.[42] Our results are concomitant with another study done in Tanzania where 84% of patients with acute respiratory infection were sold one antibiotic on request.[43] This practice explains how low the pharmacy retailers’ level of competency is. A competent pharmacy retailer would not honor such prescriptions and therefore would not dispense any antibiotic for runny nose. Antimicrobials are overused due to poor adherence to TFDA’s dispensing rules and regulations, poor public health facility antibiotic prescribing, and inappropriate public demand.[43,44]

Metronidazole was dispensed for acute waterly diarrhoea by a proportion of 100% from both parts I and II pharmacies. Our findings are similar to those of another report in Nigeria[45] where metronidazole was preferentially chosen for non-prescription acute waterly diarrhoea treatment. Management of a two days’ diarrhoea without fever, blood, tenesmus, mucus, pus, nausea or vomiting is supportive care with fluids and electrolyte replacement.[45,46] Antibiotics are not indicated in the treatment of acute waterly diarrhoea.[47] It is unfortunate that metronidazole is perceived to manage acute waterly diarrhoea, a myth held by many clients and dispensers alike.[48] In a study from Moshi, Tanzania 80.6% of children with diarrhea had antibiotic prescription[49] indicating indiscriminate antibiotic use in diarrhoea treatment instead of fluid and zinc replacement therapy. This further underscores the fact that irrational prescription practice is deep rooted and requires an equally massive educational campaign among community pharmacy retailers.

Fever was treated by antibiotics without prescriptions by a proportion of 100% using trimethoprim—sulphamethoxazole from part II pharmacies and with prescriptions by azithromycin and erythromycin from part II pharmacies. While it was inappropriate to sell and dispense antibiotics with or without prescription for fever, we sought to test the retailers’ dispensing competency, which we found it to be poor. Fever usually connotes an infective aetiology. A fever study in northern Tanzania showed the main fever aetiology among hospitalized patients to be leptospirosis by 33%; while bacterial cause was 9.8%.[50] It follows that antibiotic dispensing for fever is largely inappropriate but unfortunately a routine behavior among clinicians and drug retailers. We expected the retailers to refuse dispensing antibiotics on grounds of inappropriateness of the antibiotic use. The complacency shown by the retailers connotes non—adherence to the TFDA’s dispensing rules and regulations.

The proportion of retailers dispensing antibiotics without instructions for use in part I pharmacies was high (85%). Antibiotic dispensing without instructions may lead to wrong self-dosages and inappropriate dosing intervals that might harm the users. Similar findings are evident in Asia[16] where instructions for medicine use was very minimal, if any, and had to be probed by clients. One study has attributed a lack of instructions for medicine use to poor retailers’ knowledge and training[51] in LMICs. This is a potential area for intervention.

All retailers had to be probed for antibiotic side effects. Many retailers usually do not discuss with their clients about antibiotic side effects.[52] Our results are similar to an Indian study where no explanation was given on the side effects and potential drug allergies.[48] This problem is also present in tertiary care hospitals.[53] An intervention is needed to address this problem.

An incomplete dose i.e. an oral two days’ course of antibiotics was dispensed in almost all encounters (71.4%). We expected the dispensers to refuse dispensing an incomplete course of antibiotics. However, they did not and proceeded to dispense an incomplete antibiotic dose. An oral two days’ course of antibiotics is clearly a sub-optimal antimicrobial use. Sub-optimal antimicrobial use normally fails to clear the infection and may lead to antibiotic resistance.[54] One report showed an evidence of imipenem resistance due to short duration of antibiotic therapy.[55] In Dar es Salaam, Tanzania dispensing of incomplete oral antibiotic dose was reported to be 30% in private pharmacies from 1573 medicines dispensed in 2011.[56] In a country where majority of population rely on community pharmacies as first point of health care this should raise a cautious alarm on antibiotic misuse. Our data show that 5 (50%) incomplete antibiotic doses were dispensed in part I while 12 (54.6%) were dispensed in part II pharmacies. This connotes that six years down the line the practice is still ongoing. Measures to curb this practice by pharmacy licensing authorities in Tanzania are deemed necessary. A study indicated that factors responsible for the dispensing incomplete antibiotic doses in LMIC include economic constraints, a desire to test the therapeutic efficacy and presence of side-effects before purchasing larger quantities, and a belief that a full course is unnecessary.[16] All these factors do not, in our opinion, justify the dispensing incomplete antibiotic dose practice owing to the looming consequence of antibiotic resistance. Currently there is a debate on the appropriate duration of an antibiotic course. It is argued that the severity of infection may be a good indicator of the antibiotic duration.[57] Majority of guidelines recommend a 5–7 days antibiotic course to avoid “antibiotic selection pressure” as the mechanism of development of antibiotic resistance.[58] A delicate balance exists between supplying an adequate antibiotic dosing duration to clear an established infection at the same time minimizing destruction of the body’s protective normal flora. One study suggests monitoring infection through procalcitonin, but this is impractical in LMIC[57]. Henceforth, de-escalation from intravenous, broad spectrum to oral, narrow spectrum antibiotic is advocated although it is a challenge due to poor diagnostic capacity in LMIC.[59,60]

In Tanzania and elsewhere in LMICs, drivers of non-prescription antibiotic dispensing behaviors include community pharmacies prioritizing profit making over a good pharmacy practice,[30] dispensers’ behavior being driven by customer request,[61,62] habit (“mazoea”) following inappropriate health facility prescriptions, the need to make a profit [63] and fulfillment of clients expectations.[64] All these drivers significantly contribute to inappropriate community antimicrobial consumption, a risk factor for development of community antimicrobial resistance.

This study commands several methodological strengths. Firstly the Simulated Clients Method depicts the actual practice and behavior since retailers are not aware of the study and are made to believe they are treating real patients. Secondly, part I and part II community pharmacies are an ideal avenue to observe and describe the actual non-prescription dispensing behavior indicating community antibiotic consumption. We acknowledge several limitations of this study. First, the study was not able to collect data on retailers’ qualifications. Retailers’ qualifications would have indicated the level of their competency and we could be able to clearly associate the observed dispensing behavior. But on other hand collecting retailers’ qualifications would influence the dispensing behaviors. Second, we studied a single town only, which warrants a cautious interpretation of these findings. For generalizability of the findings, data from other urban areas of Tanzania need to be collected and collated.

## Conclusions

We have identified a widespread antibiotic dispensing practice from community pharmacies and drug stores without prescription in Moshi, Tanzania. The readily accessibility of antibiotics may lead to inappropropriate antibiotic consumption, a breeding ground for antibiotic resistance. We have identified weakness in antibiotic dispensing where retailers neither give instructions for medicine use nor do they explain side effects. We thus recommend mass health education campaign to retailers and the public at large. Secondly, we have observed a lack of observing prescription—only rules and regulations while dispensing antibiotics. We recommend regular inspections by TFDA at community pharmacies and drug stores to check adherence to prescription—only antibiotic dispensing policy and practice. A strong legislative component to prescription only policy on antibiotics may not be over-emphasized given the current extent of the vice in Tanzania. An urgent need to initiate community antibiotic stewardship awareness and antimicrobial stewardship programmes training among clinicians who prescribe is paramount so as to reduce the volumes of antibiotic consumption to mitigate the risk of development of community antimicrobial resistance.[65] Prescribers should be obliged to follow national guidelines for antibiotic prescription. Pharmacists and pharmacy owners are liable to adhere to laws and regulations governing antibiotic dispensing. Continuing professional development and on job training to drug dispensers should be routinely emphasized. Future studies should seek to know the pharmacy dispensers’ qualifications, which was beyond the scope of this study. A further research endeavor should establish retailers’ knowledge on the effects and consequences of antibiotic non-prescription practice.

## Supporting information

S1 Questionnaire (DOC)
