# Supplementary material for: Prescription and non-prescription antibiotic dispensing practices in part I and part II pharmacies in Moshi Municipality, Kilimanjaro Region in Tanzania: A simulated clients approach
Source: PLoS One. 2018 Nov 21;13(11):e0207465. doi: 10.1371/journal.pone.0207465 (PMC6248976; doi:10.1371/journal.pone.0207465)
Supplement: S1 Questionnaire — (DOC) [file pone.0207465.s001.doc]

1. Questionnaire No
2. Pharmacy category: **○** Part I **○** Part II
3. Prescription given? **○** No **○** Yes
4. Dose type: **○** Complete **○** Incomplete
5. Symptoms:

Cough  Diarrhoea

Runny nose  Fever

Pain urination

1. Prescriptions:

Ceftriaxone  Ciprofloxacin

Loperamide  Azithromycin

TMX/SMX  Metronidazole

Amoxyclav  Paracetamol

1. Medication given:

**○** No

**○** Yes

- 1. If Yes, name of drug1:

|  |
| --- |

Name of drug 2:

|  |
| --- |

Name of drug 3:

|  |
| --- |

1. Reasons if medication not given (***skip 8 if 7 is Yes***):

**○** No Prescription

**○** Not Available

**○** Wrong indication

1. Alternative medication given:

**○** No

**○** Yes

- 1. If Yes, name of drug1:

|  |
| --- |

Name of drug 2:

|  |
| --- |

Name of drug3:

|  |
| --- |

1. Reasons given for alternative medication (***skip 10 if 9 is No***):

**○** No

**○** Yes

1. Instructions for medicines use:

**○** Voluntarily

**○** After probing

1. Side Effects explained:

**○** Voluntarily

**○** After probing

1. Antibiotics given for 2 days instead of 7 days (***Skip 13 if dose type is complete*)**

**○** No

**○** Yes

1. Reasons for not giving antibiotics for 2 days: **(*Skip 14 if dose type is complete***)

Insufficient money

Incomplete dose not allowed
